# Methicillin-resistant *Staphylococcus aureus* in China: a multicentre longitudinal study and whole-genome sequencing

**DOI:** 10.1080/22221751.2022.2032373

**Published:** 2022-02-10

**Authors:** Bingjie Wang, Yanlei Xu, Huilin Zhao, Xinyi Wang, Lulin Rao, Yinjuan Guo, Xie Yi, Longhua Hu, Shuying Chen, Lizhong Han, Junying Zhou, Guoxiu Xiang, Long Hu, Liang Chen, Fangyou Yu

**Affiliations:** a Department of Clinical Laboratory, Shanghai Pulmonary Hospital, School of Medicine, Tongji University, Shanghai, People’s Republic of China; b Shanghai Key Laboratory of Tuberculosis, Shanghai Pulmonary Hospital, Tongji University School of Medicine, Shanghai, People’s Republic of China; c Jiangxi Provincial Key Laboratory of Preventive Medicine, School of Public Health, Nanchang University, Nanchang, People’s Republic of China; d Department of Laboratory Medicine, Wenzhou Medical University, Wenzhou, People’s Republic of China; e Department of Laboratory Medicine, West China Hospital, Sichuan University, Chengdu, People’s Republic of China; f Department of Jiangxi Provincial Key Laboratory of Medicine, Clinical Laboratory of the Second Affiliated Hospital of Nanchang University, Nanchang, People’s Republic of China; g Department of Laboratory Medicine, The First Affiliated Hospital of Wenzhou Medical University, Wenzhou, People’s Republic of China; h Department of Laboratory Medicine, Ruijin Hospital, Shanghai Jiao Tong University School of Medicine, Shanghai, People’s Republic of China; i Zhongnan Hospital of Wuhan University, Wuhan, People’s Republic of China; j Department of Laboratory Medicine, The First Affiliated Hospital, Sun Yat-sen University, Guangzhou, People’s Republic of China; k Department of Bioinformatics, Hugobiotech, Beijing, People’s Republic of China; l Center for Discovery and Innovation, Hackensack Meridian Health, Nutley, New Jersey, USA

**Keywords:** Methicillin resistant, *Staphylococcus aureus*, molecular epidemiology, genome sequencing, China

## Abstract

The aim of this study was to investigate the genomic epidemiology of MRSA in China to identify predominant lineages and their associated genomic and phenotypic characteristics. In this study, we conducted whole-genome sequencing on 565 MRSA isolates from 7 provinces and municipalities of China between 2014 and 2020. MRSA isolates were subjected to MLST, *spa* typing, SCC*mec* typing, analysis of virulence determinants and antimicrobial susceptibility testing. Among 565 MRSA isolates tested, clonal complex (CC) 59 (31.2%), CC5 (23.4%) and CC8 (13.63%) were the major lineages, and the clonal structure was dominated by ST59-t437-IV (14.9%), ST239-t030-III (6.4%) and ST5-t2460-II (6.0%), respectively. Of note, CC8, the predominant lineage in 2014–2015, was replaced by CC59 after 2016. Interestingly, the extension and unstable structure of the CC5 population was observed, with ST5-t311-II, ST764-t1084-II, ST5-t2460-II and ST764-t002-II existing complex competition. Further analysis revealed that virulence determinant profiles and antibiograms were closely associated with the clonal lineage. The CC59 MRSA was less resistant to most tested antimicrobials and carried fewer resistance determinants. But rifampicin resistance and mupirocin resistance were closely linked with CC8 and CC5, respectively. MRSA isolates conservatively carried multiple virulence genes involved in various functions. PVL encoding genes were more common in ST338, CC30, CC398, ST8 and CC22, while *tsst*-1 was associated with ST5. In conclusion, the community-associated CC59-ST59-t437-IV lineage was predominant in China, with diverse clonal isolates alternately circulating in various geographical locations. Our study highlights the need for MRSA surveillance in China to monitor changes in MRSA epidemiology.

## Introduction

Methicillin-resistant *Staphylococcus aureus* (MRSA) is a notorious multidrug-resistant bacterium, which can cause a series of infectious diseases, such as septic shock, endocarditis, and severe pneumonia [1]. MRSA infections are complicated to manage since these pathogenic bacteria are resistant to multiple antibiotics. During the past few decades, the spread of MRSA, including several clonal lineages of hospital-associated MRSA (HA-MRSA) and community-associated MRSA (CA-MRSA) and more recently livestock-associated MRSA(LA-MRSA) had been a major health problem worldwide [2]. ST239 and ST5 are the classic HA-MRSA clones prevalent in China and other Asia countries, while heterogenetic CA-MRSA clones such as ST59, ST338, ST30, ST72 and ST8 are disseminated in different regions [2,3].

Although there have been several epidemiological studies of MRSA in mainland China, they mainly focus on a subset of MRSA, such as specific infection type source or community origin [4,5]. However, MRSA infections due to lineages that were initially exclusively associated with CA-MRSA have been observed in hospital settings with increasing frequency, which may cause the overlap of the definition [2,6]. Knowledge of the entire MRSA population in hospital settings in China was still limited.

Analysing the genotypic characteristics of MRSA clones is valuable for understanding MRSA evolution and dissemination [1,7]. Whole-genome sequencing allows for a comprehensive analysis of the epidemiology and genomic repertoire of *S. aureus* and provides in-depth insights into the evolution of particular MRSA clones [6,8]. While few studies have characterized extensive collections of MRSA isolates from China using whole-genome sequencing [9,10].

Untargeted profiling of the entire MRSA population in a specific area is crucial in monitoring its emergence and spread and informing prevention strategies [11]. To produce a more comprehensive national description of the molecular epidemiology and phenotypic properties of MRSA in China, we characterized 565 MRSA isolates collected from several geographically dispersed Chinese hospitals over the past seven years.

## Materials and methods

### MRSA isolates

**Figure 1.**
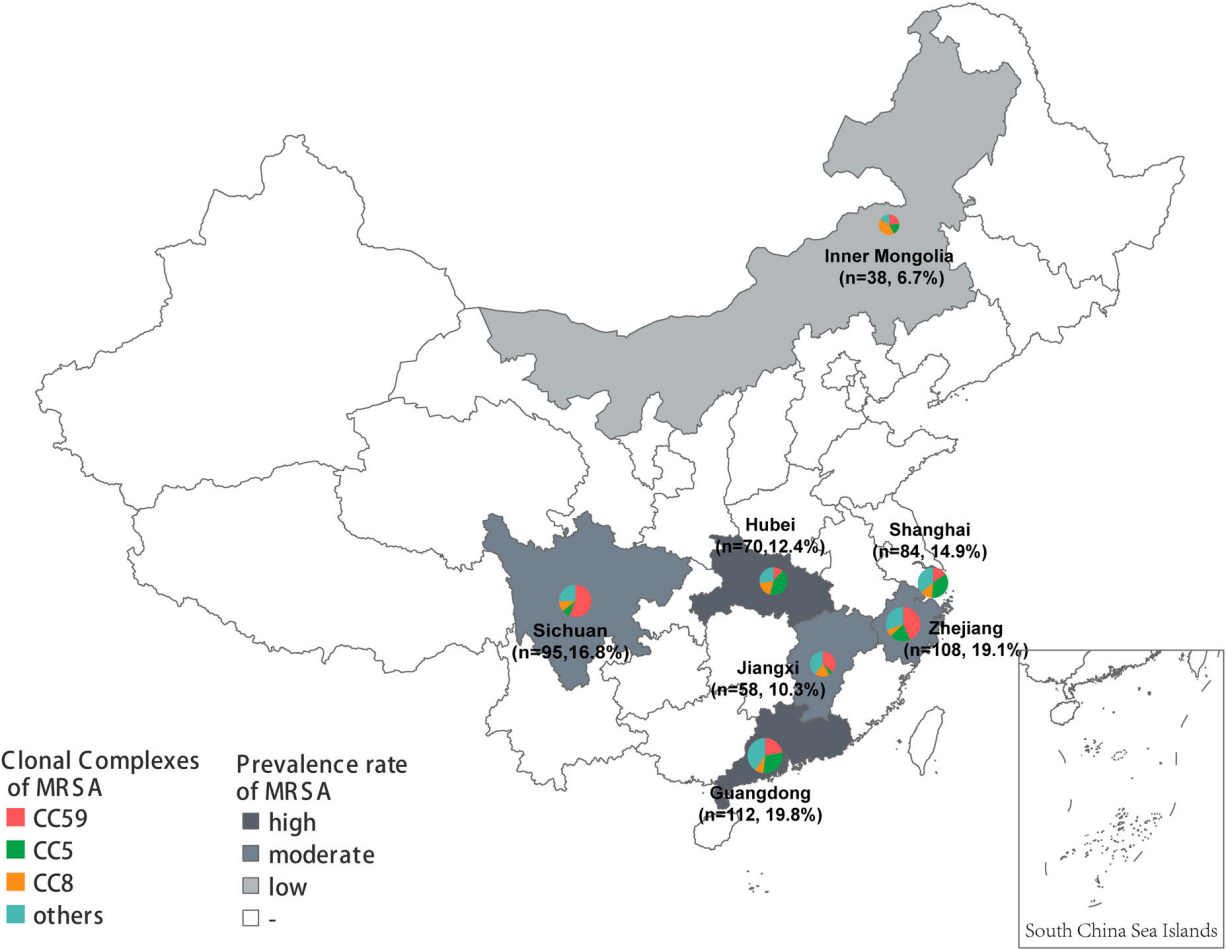
The geographical distribution of 565 MRSA clinical isolates in this study. The circle size represents the number of isolates, and circle partitions represent the prevalence of different CCs of MRSA recovered from separate locations. The prevalence rate of MRSA for each location was adopted from the China Antimicrobial Resistance Surveillance Report (http://www.carss.cn/Report).

For this study, non-duplicated MRSA clinical isolates were collected between 2014 and 2020 from 7 provinces and municipalities in China, including Shanghai, Zhejiang, Guangdong, Sichuan, Hubei, Jiangxi, and Inner Mongolia. These regions represented varying prevalence levels of MRSA in China [12] (Figure 1). One representative tertiary care hospital was selected in each region for further investigation. In all the stored MRSA from 2004 to 2020, chosen randomly MRSA using random selection module in Microsoft excel. Information about these isolates was extracted from the laboratory information system. All isolates were reidentified species by the MALDI-TOF MS (Bruker Daltonics GmbH, Bremen, Germany), and cefoxitin resistance was determined by the disc diffusion method [13].

## Antimicrobial susceptibility testing

A total of 18 antimicrobial agents were tested for antimicrobial susceptibility. Erythromycin, clindamycin, quinupristin-dalfopristin, ceftaroline, ciprofloxacin and tetracycline were tested by the disc diffusion method in Mueller–Hinton agar plates (Oxoid, UK). The minimal inhibitory concentrations (MICs) of cefoxitin, oxacillin, gentamicin, fusidic acid, mupirocin, rifampicin, sulfamethoxazole/trimethoprim, daptomycin, teicoplanin, linezolid, vancomycin, dalbavancin (Sigma-Aldrich, United States) were determined using the broth microdilution method [13]. D-test was performed to determine inducible clindamycin resistance. *S. aureus* ATCC25923 and ATCC29213 were used as quality control organisms. Interpretative criteria were consistent with the CLSI document, except for fusidic acid, for which breakpoints from EUCAST were applied [13,14].

## Genome sequencing and bioinformatic analyses

Total DNA from the MRSA isolates was extracted using QIAamp DNA Mini Kit (Qiagen) and sequenced on the Illumina NovaSeq platform using the 2 × 150–base pair paired-end mode. The raw reads were trimmed, and *de novo* assembled into contigs using CLC Genomics Workbench software (version 12.0; CLCbio). The average sequencing depth was 224.3 ± 57.92. An average of 4.34 ± 1.18M reads count was obtained from each MRSA isolate, and the GC rate was 32.72 ± 0.05. The contig number was 49.73 ± 59.57, with 217.9 ± 159.8Kbp of N50.

The assembled contigs were used for molecular typing, including MLST, *spa*-typing and SCC *mec* typing. STs were inferred using the *S. aureus* MLST database (https://pubmlst.org/organisms/staphylococcus-aureus). Moreover, the *spa* and SCC*mec*-types were predicted using the Centre for Genomic Epidemiology website (https://cge.cbs.dtu.dk/services). In addition, the phylogenetic relationship of all MRSA genomes was analysed using recombination-filtered core SNPs by ParSNP [15].

Furthermore, the WGS data were also used for identifying the virulence factor genes by ABRicate v1.01 (https://github.com/tseemann/abricate) using VFDB database [16], and candidate genes were associated with adhesion, exoenzymes, IEC, immune evasion, secretion, toxin, haemolysis, biofilm formation and iron uptake and metabolism [17].

## Results

### MRSA strains and their antimicrobial susceptibility

**Figure 2.**
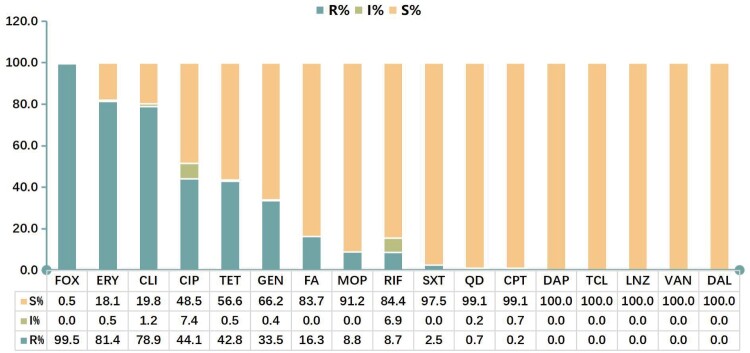
Antimicrobial susceptibility of MRSA strains. FOX, cefoxitin; OXA, oxacillin; ERY, erythromycin; CLI, clindamycin; CIP, ciprofloxacin; TET, tetracycline; GEN, gentamicin; FA, fusidic acid; MOP, mupirocin; RIF, rifampicin; SXT, sulfamethoxazole/trimethoprim; QD, quinupristin-dalfopristin; CPT, ceftaroline; DAP, daptomycin; TCL, teicoplanin; LNZ, linezolid; VAN, vancomycin; DAL, dalbavancin.

A total of 565 non-repetitive clinical MRSA isolates were collected from the outpatients and inpatients in seven hospitals, as shown in Figure 1. They were isolated from blood (34.5%, 195/565), pus/wound exudate (31.5%, 178/565) and sputum (34.0%, 192/565) of patients. All MRSA isolates carried *mecA* gene, while 3 strains (0.5%) were susceptible to cefoxitin, and 7 stains (1.2%) were susceptible to oxacillin. Resistance to erythromycin (81.4%) and clindamycin (78.9%) was very common. The resistance rates to ciprofloxacin, tetracycline and gentamicin were 44.1%, 42.8% and 33.5%, respectively. Fusidic acid, mupirocin, rifampicin, sulfamethoxazole – trimethoprim, quinupristin-dalfopristin and ceftaroline remained active against the majority of MRSA with a resistance rate of 0.2–16.3%. Additionally, none of the MRSA isolates were resistant to daptomycin, teicoplanin, linezolid, vancomycin or dalbavancin, indicating these antibiotics had superior antimicrobial activity against MRSA isolates **(**Figure 2 and Figure S1).

## Molecular typing characteristics of MRSA isolates

**Figure 3.**
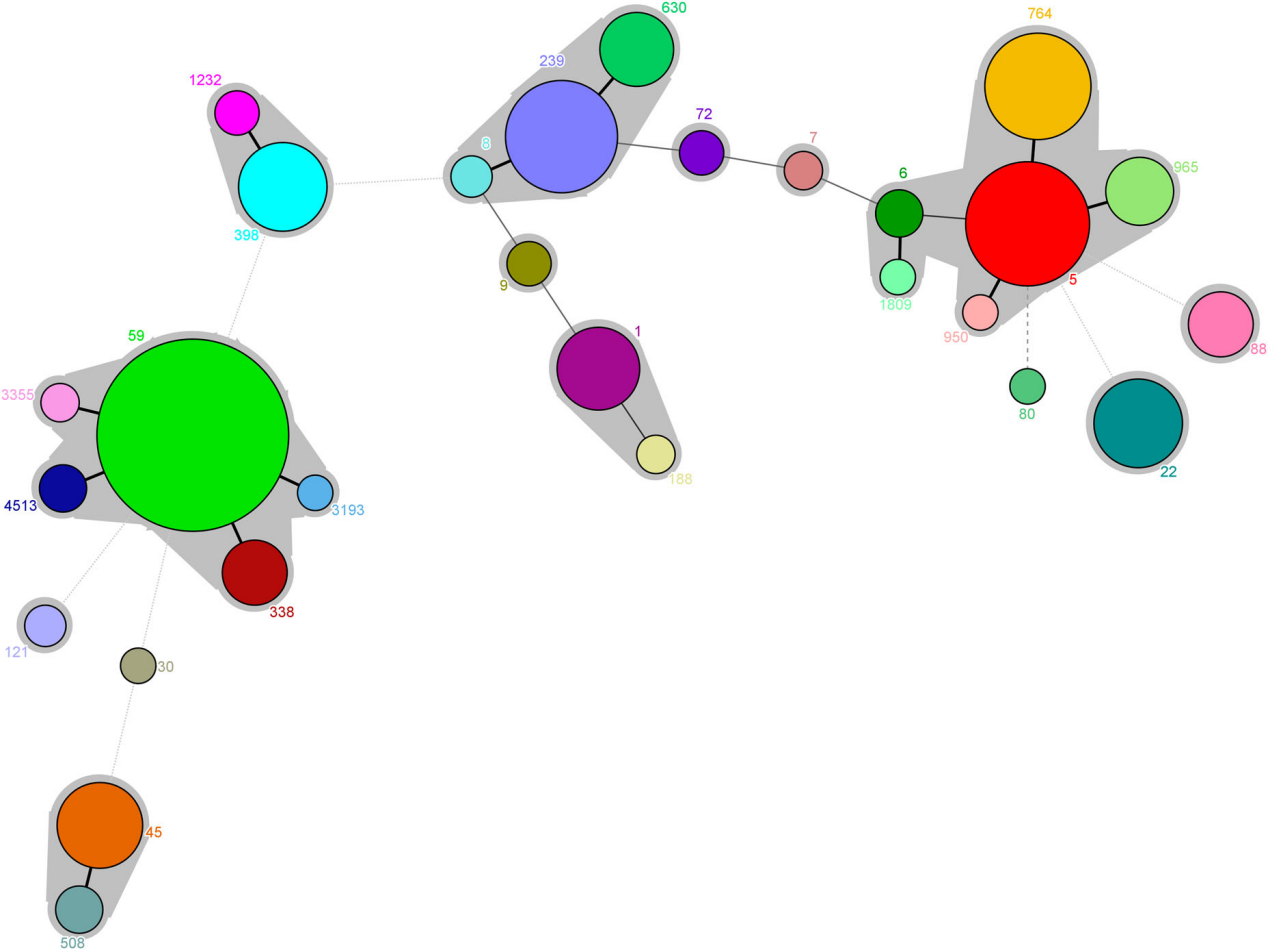
Minimal spanning tree based on the multi-locus sequence types of MRSA strains. All 28 STs are represented by a circle with colour. The size of each circle indicates the number of isolates of specific types. The shaded halo surrounding the STs encompasses related STs that belong to the same CC. These non-typable isolates were not included in this analysis.

Among 565 MRSA isolates, 28 distinct STs were identified, with ST59 (27.1%, 153/565), ST5 (11.0%, 62/565), ST239 (9.0%, 51/565), ST764 (8.1%, 46/565), ST398 (5.3%, 30/565) most frequently encountered (Figure 3). Thirty-one MRSA isolates belonged to novel STs. Twenty-eight STs identified were assigned to 14 clonal complexes (CCs). One CA-MRSA lineage CC59 (31.2%, 176/565) and two HA-MRSA lineages CC5 (23.4%, 132/565) and CC8 (13.6%, 77/565) were most frequent (Figures 3 and 7). Of note, unlike CC59 and CC8 clones often isolated from blood or pus/wound exudate, most of CC5 was isolated from sputum (Table S1).

The *spa* typing discriminated 565 isolates into 93 types, and t437 was the most frequently represented type (20.2%, 114/565), followed by t2460 (8.0%, 45/565), t30 (6.4%, 36/565). Notably, 114 t437 isolates belonged to CC59 and all 21 t034 isolates were classified as CC398 (Table S1).

By SCC*mec* typing, six types (IV, II, V, III, XII, VII) were found within the 565 isolates. SCC*mec* IV was the most predominant type, accounting for nearly half of all MRSA isolates (48.8%, 276/565). This cassette often associated with CC59 (ST59 and its variant ST4513) and several CC5 lineages (ST965 and ST6). SCC*mec* II was the second most cassette (21.2%, 120/565) and strongly associated with the New York/Japan clone ST5 and its variant ST764. SCC*mec* V (12.7%, 72/565), SCC*mec* III (9.4%, 53/565) were also abundant, and most of SCC*mec* III belonged to ST239. Thirty-nine isolates were not assigned to any SCC*mec* type, and most of them (59.0%, 23/39) belonged to ST398 (Table S1). Among them, twenty-seven isolates were harboured a class C2 *mec* complex and one copy of *ccrC*1, one isolates were contained *mecA-mecR1-mecI* structure, while other 12 isolates were only identified *mecA* gene using SCC *mec* Finder.

With all the molecular typing data combined, the clonal structure of the MRSA isolates in our study was dominated by the Asian-Pacific clone CC59-ST59-t437- IV (14.9%, 84/565), the Eurasian clone CC8-ST239-t030-III (6.4%, 36/565) and the New York/Japan clone CC5-ST5-t2460-II (6.0%, 34/565) [18].

## Comparison of antimicrobial resistance pheno- and genotypes of MRSA major clonal complexes

**Figure 4.**
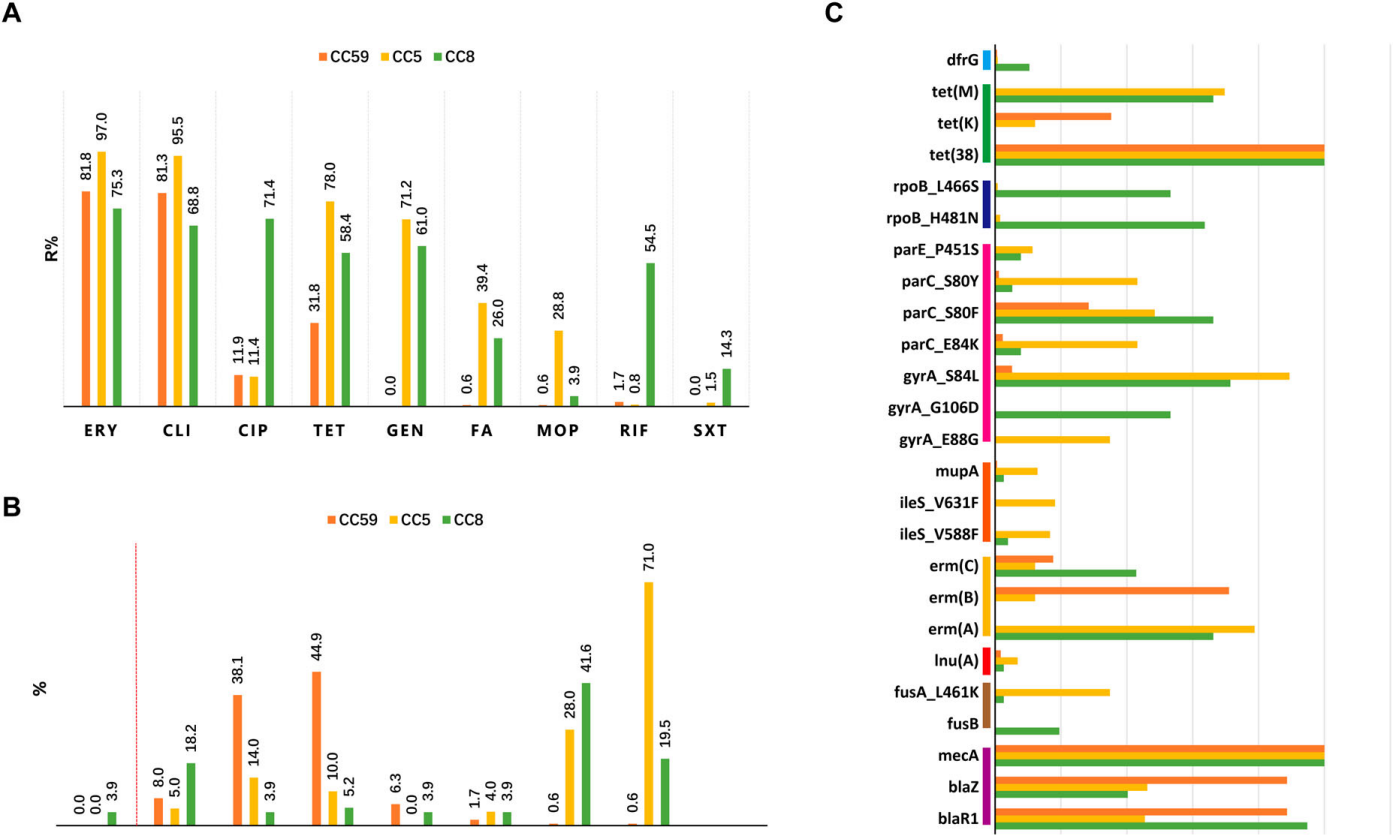
Antimicrobial susceptibility profiles among different CCs of MRSA isolates. (A) Resistance rates to various antimicrobials. These antimicrobials with <1.0% overall resistance rate were not included in this analysis. (B) Methicillin MIC distribution. The ≥8 MIC of methicillin was defined as methicillin resistance. (C) The resistome analysis. Only resistance determinants found in more than 10 isolates and carried by CC59, CC5, or CC8 are specifically displayed. For more details, see Table S2.

The dominant CCs MRSA isolates, including 176 CC59, 132 CC5 and 77 CC8 isolates, displayed various antimicrobial resistance profiles, as shown in Figure 4 and Table S2,. Overall, CC59 displayed low resistance for most tested antimicrobial agents, except for erythromycin and clindamycin, as for the less content of numerous resistance determinants. Specifically, the resistance rate of the CC59 isolates to tetracycline was significantly lower than the CC5 and CC8 isolates (31.8% *vs* 78.0% and 58.4%, *p *= 0.000). Resistance to gentamycin, fusidic acid and mupirocin were rarely identified in the CC59 isolates (0.0%∼0.6%), whose genomes tended to lack *aac (6’)-Ie/aph (2’’)-Ia*, *fusA*_L461 K, *fusB*, *mupA, ileS_*V631F and *ileS_*V588F (Figure 4(A,C)). Moreover, unlike CC5 or CC8 with high cefoxitin resistance level (the most frequently observed MICs were ≥256 mg/L), the distribution of cefoxitin MICs of CC59 was mainly concentrated in 16 mg/L or 32 mg/L (Figure 4(B)). Notably, CC8 isolates tend to be resistant to rifampicin, of which resistance mostly conferring by *rpoB*_L466S and *rpoB*_H481N; CC5 isolates more likely to be resistant to mupirocin, and higher carriage rates of *mupA* and *ileS* mutations (Figure 4(A,C)).

## Prevalence characteristics of MRSA in different geolocations and periods

National surveillance data from CRASS (China Antimicrobial Resistance Surveillance System) showed that the median prevalence rate of methicillin resistance in *S. aureus* during 2014–2019 was 23.1–46.9% in different provinces of China [12]. Regarding the 565 MRSA isolates in this study, variations in the prevalence rate of varying predominant MRSA clonal complexes was observed in diverse geographical locations. CC5 was more inclined to establish local endemic in regions with high MRSA morbidity rates, including Guangdong (29.5%, 33/112), Shanghai (35.7%, 30/84) and Hubei (42.9%, 30/70); CC59 was more prevalent in areas with a moderate incidence of MRSA, including Sichuan (55.8%, 53/95), Jiangxi (36.2%, 21/58) and Zhejiang (43.5%, 47/108). In contrast, in Inner Mongolia with a low MRSA prevalence rate, CC8 was the most common genotype (42.1%, 16/38) (Figure 1).

**Figure 5.**
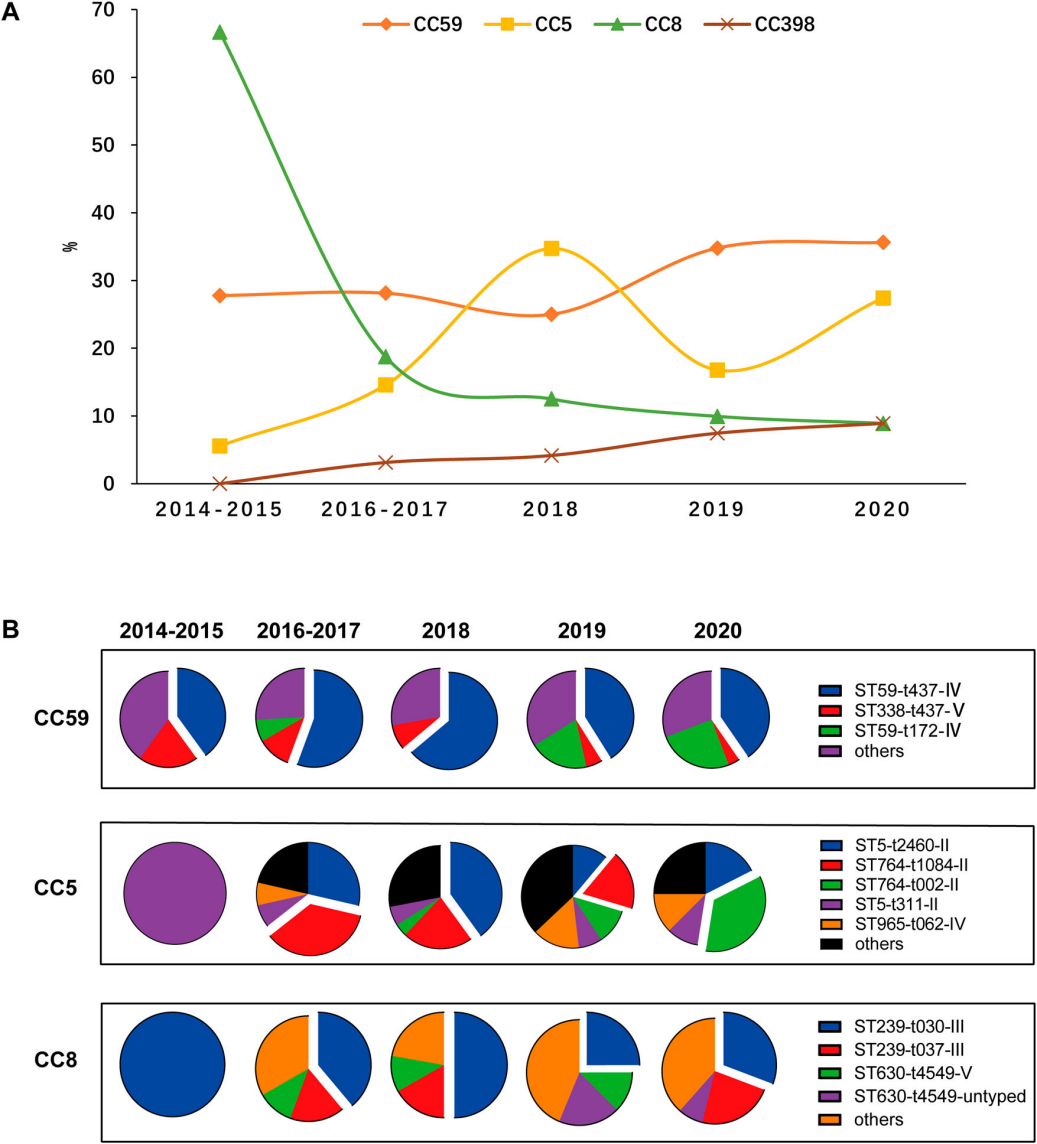
Major CCs found in MRSA isolates by years. (A) The distribution of four CCs over time. (B) The clonal structure of CC59, CC5 and CC8 in different years.

Additionally, clonal replacement of predominant MRSA strains was observed in this study (Figure 5(A)). The CC8 lineage (mainly ST239-t030-III) was overwhelmingly predominant in 2014–2015 with up to 66.7% frequency, while subsequently losing its superiority and dramatically declining to 8.9% in 2020. Meanwhile, the frequency of CC59 (mainly ST59-t437-IV) and CC5 (mainly ST5-II and ST764-II) exhibited an escalating trend with from 27.8% in 2014–2015 to 35.6% in 2020 and 5.6% in 2014–2015 to 27.4% in 2020, respectively (Figure 5(A,B)). Also it is worthy to notice that CC398, a notorious livestock-associated lineage in European countries, Australia, North and South America, increased from 0% in 2014–2015 to 8.9% in 2020 [19].

## Dynamic population structure of CC5

There was an association between CC5 and the high MRSA morbidity (Figure 1). In the current study, CC5, primarily composed of New York/Japan clone ST5 and its variant ST764, displayed a jagged increase trend over the past seven years (Figure 5(A,B)). Interestingly, we also noticed that the multiple lineages in this CC population involved replacement events along with this trend, including ST5-t311-II, ST764-t1084-II, ST5-t2460-II and ST764-t002-II (Figure 5(B)). This temporal dynamic of heterogeneous clones indicated the potential for more complex competitive interactions in CC5. Moreover, although the geographical distribution of CC5 was scattered, ST5-II was more frequent in Hubei (45.2%, 28/62), and ST764-II exclusively prevalent in coastal cities, including Shanghai (47.8%, 22/46) and Guangdong (41.3%, 19/46) (Table S1).

## Phylogenetic analysis based on core genome SNPs of STs

**Figure 6.**
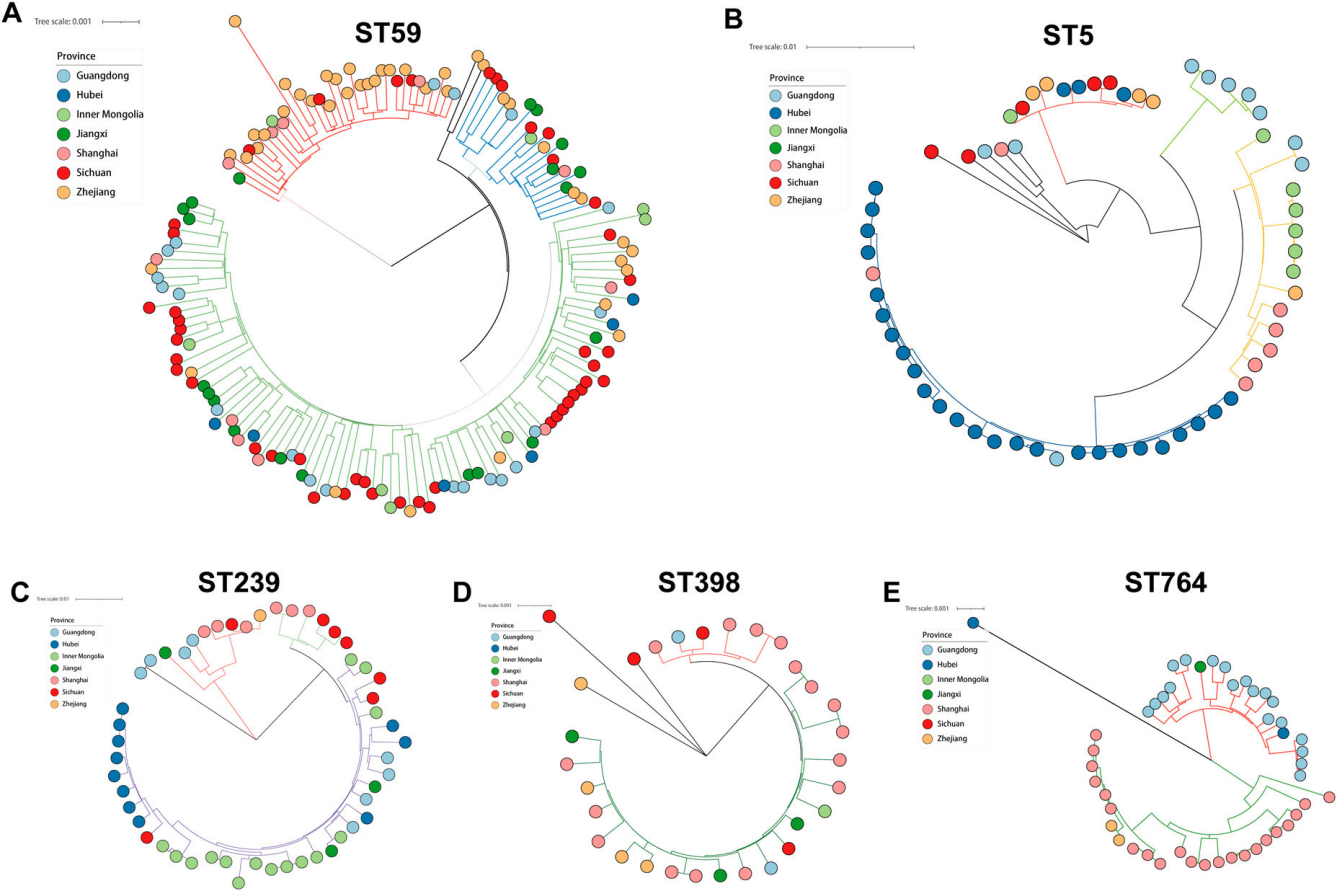
Core genome phylogenies for ST59 (A), ST5 (B), ST239(C), ST398, and ST764 (E). Phylogenetic trees were based on 88.4–89.8% core genome and 11844–47901 SNPs. The geographical origin of MRSA isolates in this study (circle symbol) is indicated with colours. Phylogenetic clades are indicated by the colours of branches.

The phylogenetic trees of specific STs, including ST59, ST5, ST239, ST764 and ST398, were generated to investigate their heterogeneity further (Figure 6). Interestingly, all these STs were clustered into two or more clades, indicating their high diversity. ST59 frequently transmit between sampled provinces, while ST239, ST5 and ST764 clones often spread locally. This was supported by the fact that ST59 clones from various provinces were interspersed in phylogenetic branches of ST59, while ST239, ST5 and ST764 within a single hospital setting or province tend to cluster into tightly linked clades.

We expanded these datasets to include genomes from publicly available worldwide collections (Figure S2). The global phylogenetic analysis of strains showed that ST59, ST5, and ST239 in China and European countries or North America tend to fall into different evolutionary clades. But, this pattern was not observed for the ST398 clone. China (*n* = 90), France (*n* = 20) and USA (*n* = 6) ST398 MRSA and MSSA strains clustered in the same clade, which also includes ST398 strains from Latin America and other European countries. Among them, the global pandemic ST5 clone represented the most heterogeneous cluster with at least fifteen clades. Notably, as a single-locus variant of ST5, ST764 has almost no publicly available sequenced genome, but it has established a local endemic in China.

## Distribution of virulence determinants

In this study, 81 virulence factors were analysed for all these 565 isolates. Half of the analysed genes (40/81) associated with capsule, exoenzymes, secretion, iron uptake and metabolism, biofilm formation and haemolysis were highly conserved and prevalent in almost all strains (≥99%) (Table S3).

**Figure 7.**
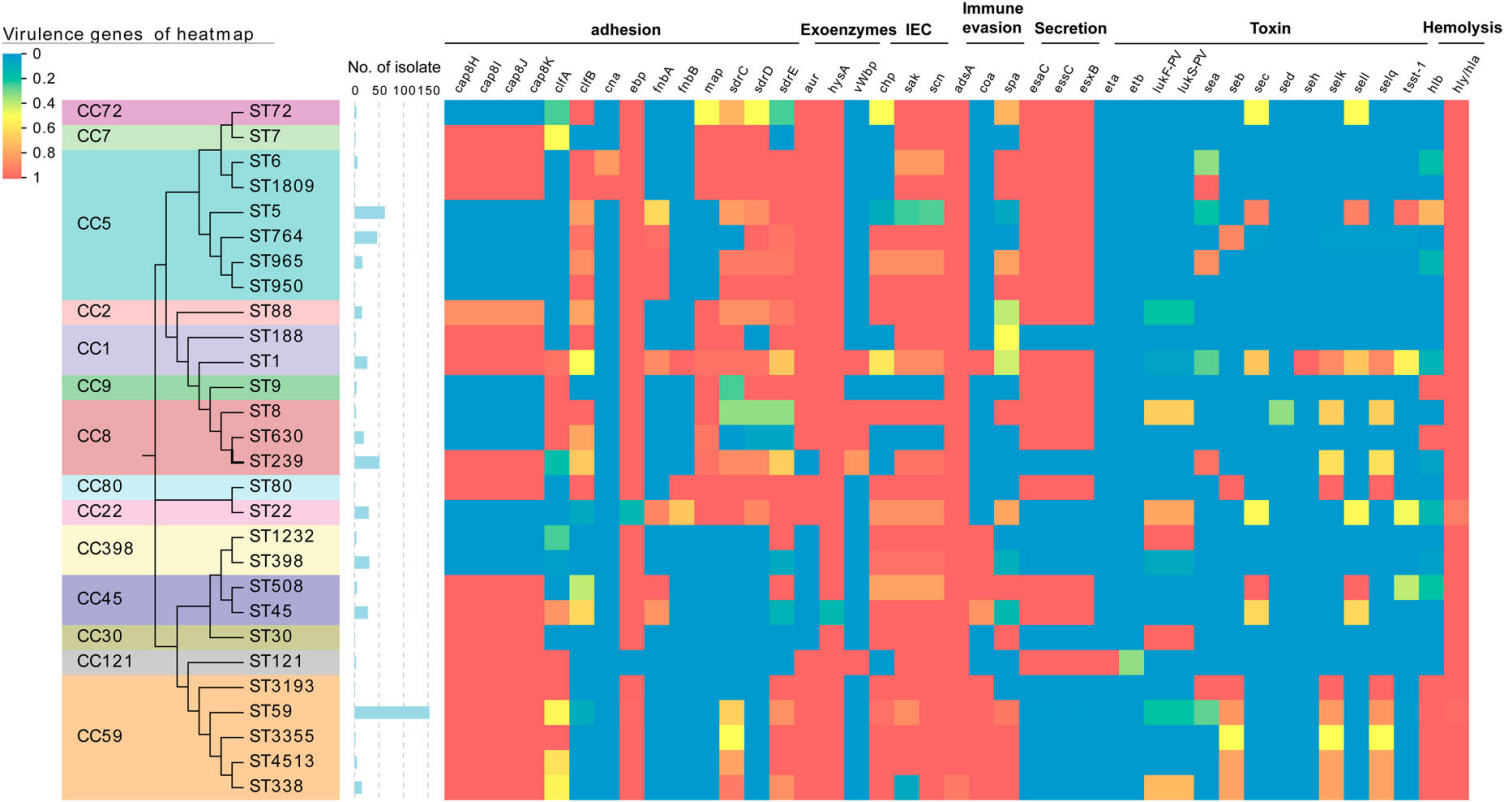
Heatmap illustrating virulence genotype of different STs (CCs). The evolutionary tree based on MLST was generated in MEGA X using the Maximum Likelihood method and Tamura-Nei model with 1000 bootstrap replicates. These virulence factor genes with more than 99% carriage were not displayed in this figure. For more details, see Table S3.

The MRSA strains showed a diverse repertoire of virulence determinants across different lineages (Figure 7). Typical human IEC (immune evasion cluster) was usually carried by ΦSa3 integrated into the *hlb* gene, and often a loss in host shift from human to animals [20,21]. IEC associated *scn*, *sak*, *chp* were detected in most isolates of STs in our cohort but absent in CC9 and ST630, indicating their potential livestock association. It’s worth noting that the *hlb* and IEC (*scn*) were alternately identified in most isolates due to the insertion of ΦSa3 in *hlb*, except CC59 harboured both of them, which might indicate the uniqueness of IEC in CC59.

The incidence rates of toxin encoding genes were only 0.2–37.2%, while 446 out of 565 isolates possess at least one toxin gene (Figure 7 and Table S3). Regarding Panton-Valentine leucocidin (PVL), only 14.3% of isolates harboured *pvl* (*lukF-PV* and *lukS-PV*). Most CA-MRSA ST59 clones lacked *pvl*, while a high frequency of this gene was observed in ST338, ST8, ST1232, CC22, and CC30 (>66.7%). Toxic shock syndrome toxin encoding gene *tsst*-1 was identified in most ST5 isolates (96.8%) and some strains of ST508, ST1, and CC22 with a low frequency of 40.0–53.8%. Additionally, the adhesion associated with *fnbA* and *sdr* was more frequently carried by CC5 than CC59 and CC8. Exfoliative toxin encoding genes *eta*/*etb* were extremely rare and only detected in CC121. Additionally, patterns of enterotoxin genes are highly variable among MRSA isolated, while they are often associated with particular clonal lineage. Most STs of CC59 harboured *seb*-*selk*-*selq*, the majority of ST239 carried *sea-selk-selq*, while ST5 often had *sec-sell*. None of the enterotoxin encoding gene was found in ST950, CC2, CC9, CC398, CC30, CC121, ST630, ST188 and CC7.

## Discussion

Our study aimed to gain epidemiology data on the genetic and phenotypic traits of MRSA circulating in China over a period of seven years. This study presents an extensive collection of 565 MRSA isolates collected by seven provinces and municipalities covering a substantial geographical zone of the country.

In this study, CC59-ST59, CC5-ST5/ST764 and CC8-ST239 were the key lineages responsible for mediating the development of the methicillin resistance in *S. aureus* in clinical environment in China [9]. The HA-MRSA CC8-ST239 has been a pandemic linage circulating in many countries worldwide since the 1970s [22]. Previous studies and our cohort identified ST59 as the major MRSA clones across China for decades [4,23–27]. However, we confirmed the clonal shift of MRSA has occurred in Chinese hospital settings. After 2015, CC8-ST239 has lost its predominance and was replaced by CC59-ST59 and CC5. Not coincidentally, this similar clonal shift was also documented in America, Malaysia, Singaporean, Hungary, Portuguese, Czech Republic and some Latin American Countries in recent decades [2,28–30], indicating it appears to be a common phenomenon.

The finding of this work confirmed that ST59-IV became the most dominant lineage in Chinese hospitals in recent years. ST59 was one of the most successful and persistent CA-MRSA clones in Asia, initially evolved independently of hospital strains and associated with fatal infections of outside hospitals [31,32]. Our data supported previous analyses that CA-MRSA clones have switched their epidemiological niches and are the most dominant lineage in nosocomial settings [33–35].

Most CC59 in the present study lacked *pvl* and exhibited relatively low resistance to common antimicrobials [36,37]. Researchers have proved that ST59 with fewer resistance fitness costs displays more competitive, which might facilitate CC8-ST239 replaced by CC59-ST59 [38]. Moreover, several CA-MRSA clones have smaller cassette chromosome *mec* elements such as SCC*mec* IV and SCC*mec* V, which provide only low-level methicillin resistance, supporting the idea that the extended evolution of CA-MRSA lineages is generally characterized by a very low methicillin resistance level, instead of harvesting more virulence determinants [39].

Another important finding in our study is the complex competition in the CC5 population structure. We found that multiple CC5 clones, including ST5-t311-II, ST5-t2460-II, ST764-t1084-II and ST764-t002-II, were alternatively predominant many times over the past seven years. Importantly, we observed that multi-drug resistant CC5 seems to be associated with high MRSA morbidity, and most of CC5 MRSA isolates were recovered from sputum, but most epidemiological studies were solely focused on the bacteraemia associated MRSA [23,40,41]. Therefore, we speculated that the importance of CC5 in clinical settings might be underestimated. Especially, the refined temporal analysis of the different clones in our study showed that, with CC239-ST239 clones losing their dominance, the most obvious change is the marked increase in the incidence of CC5. We also found that CC5 harboured more adhesion-associated genes (*fnbA* and *sdr*) and superantigenic toxin gene *tsst*-1. However, it is still unclear whether the CC5 with an unstable clonal structure plays some potential role in the clonal replacement of CC8-ST239 in China.

Our analysis also highlights the growing public health threat of LA-MRSA clones. CC398 is associated with various animals, particularly pigs across European countries and North America. Occasionally, it can cause severe infections in humans. In our study, the incidence of CC398 persistently increased over time and displayed a high heterogeneous in the global geographical phylogenetic evaluation, suggesting that the LA-MRSA ST398 clone might be introduced in China and gain further adapt to the clinical environment.

CC9 was the livestock-associated MRSA (LA-MRSA) that prevailed prosperously in livestock farms and animals in China and other Asian nations [42]. And ST630 was also reported associated with bovine mastitis in China [43]. In our study, four ST9 and nineteen ST630 clinical isolates all had an intact *hlb* gene and no IEC genes, supported their animal origin [44,45].

Consistent with previous reports, most MRSA isolates in our study displayed multiple-drug resistance [46]. Fortunately, we confirmed that several antibiotics, such as vancomycin, teicoplanin, dalbavancin, quinupristin-dalfopristin and ceftaroline, still possessed superior antimicrobial activity and may be available in selections for anti-MRSA infections. However, intermediated resistance to vancomycin has sporadically been reported in China [38,47], alerting potential challenges in antibiotic treatment. Furthermore, our data indicated that the CC59 tends to be susceptible to multiple antibiotics in vitro, while the CC8 exclusively enriches the resistance of rifampicin. According to the surveillance data from the CHINET, resistance to quinolones, aminoglycosides and rifamycin in MRSA isolates was dramatically decreased in China recent years [48]. Our findings suggested that this shift of resistance phenotype is largely a result of clonal displacement (from CC8-ST239 to CC59-ST59).

The producer of many toxins collectively contributes to *S. aureus* virulence potential. Most of the isolates in our study carried at least one toxin gene. *pvl* was only carried by a small proportion of isolates, and there was no significant association with CA-MRSA lineage ST59 in our data. The presence of PVL has previously been strongly associated with CA-MRSA in many studies [49,50], while the findings of our study do not support this notion. Most of ST338, CC30, CC398, ST8 and CC22 carried *pvl* in the present study, demonstrating the pathogenic potential of these MRSA lineages. Although unusual in other STs, 96.8% of ST5 isolates carried *tsst*-1 gene in this study, which is consistent with the report from Suzhou, with a similarly high positive percentage (96.3%) [51]. Additionally, they emphasized that *tsst*-1 positive CC5 isolates were associated with higher mortality rates.

The limitation of this study is the absence of adequate clinical information, makes us fail to analyse the types of infection, the mortality, the baseline of patients and association with specific lineages.

This is the first longitudinal large-scale surveillance of MRSA in China, covering 7 provinces and municipalities, providing important insight into the development of MRSA. Our major findings were as follows: Although MRSA exhibiting a range of strain types were detected in China, CC59, CC5 and CC8 lineages are responsible for the spread of MRSA in China. The epidemiology of MRSA varied temporally and geographically, while CC59-ST59-t427-IV lineage continually dominance in China hospitals in recent years. Different lineages are markedly associated with specific antibiotic resistance profiles and virulence patterns.

## Supplementary Material

Supplemental Material
