# Supplementary material for: Methicillin-resistant Staphylococcus aureus in China: a multicentre longitudinal study and whole-genome sequencing
Source: Emerg Microbes Infect. 2022 Feb 10;11(1):532–42. doi: 10.1080/22221751.2022.2032373 (PMC8843102; doi:10.1080/22221751.2022.2032373)
Supplement: Supplemental Material [file TEMI_A_2032373_SM2438.zip › Suppl files/Supplemental file 3 Figure S2E.docx]

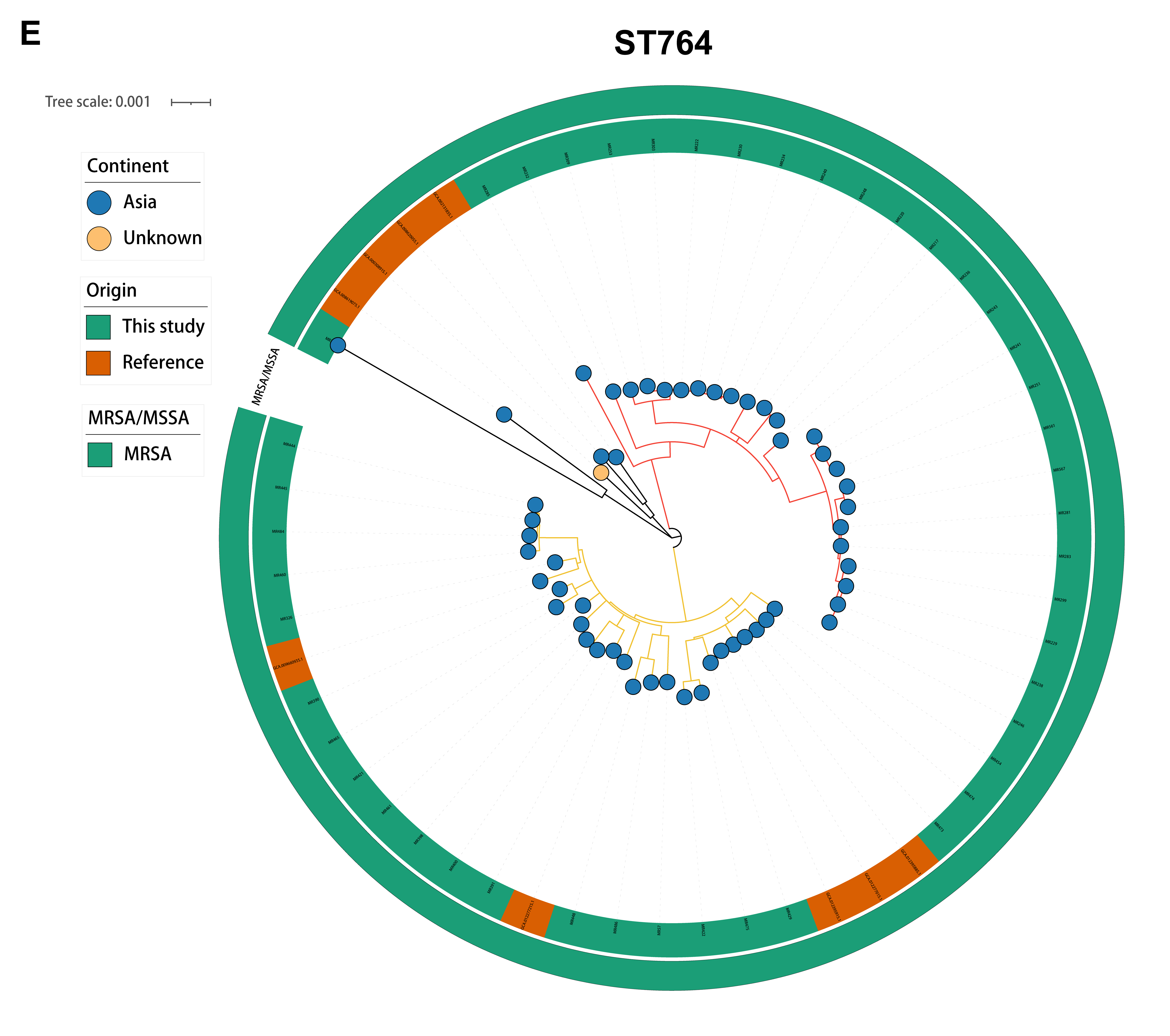


**Figure S2. Global phylogenetic trees of the five most attractive STs.** Phylogenetic clades represented by the colors of branches. The different continents are indicated by circle colors. Isolates from this study and publicly available reference genomes are color coded by the inner colored ring. Presence/Absence of *mecA* are indicated in following rings. For (A) ST59, based on 77.7% core genome and 47241 core SNPs (232 genomes from over 11 countries), (B) ST5, based on 7.6% core genome and 6401 core SNPs (2676 genomes from over 45 countries), (C) ST239, based on 68.8% core genome and 18489 core SNPs (350 genomes from over 33 countries), (D) ST398, based on 41.7% core genome and 25069 core SNPs (950 genomes from over 30 countries), and (E) ST764, based on 89.5% core genome and 20353 core SNPs (56 genomes from over 2 countries), all publicly available reference genomes in NCBI Refseq database have been included.
