# Supplementary material for: Methicillin-resistant Staphylococcus aureus in China: a multicentre longitudinal study and whole-genome sequencing
Source: Emerg Microbes Infect. 2022 Feb 10;11(1):532–42. doi: 10.1080/22221751.2022.2032373 (PMC8843102; doi:10.1080/22221751.2022.2032373)
Supplement: Supplemental Material [file TEMI_A_2032373_SM2438.zip › Suppl files/Supplemental_file_1-2_cleancopy.docx]

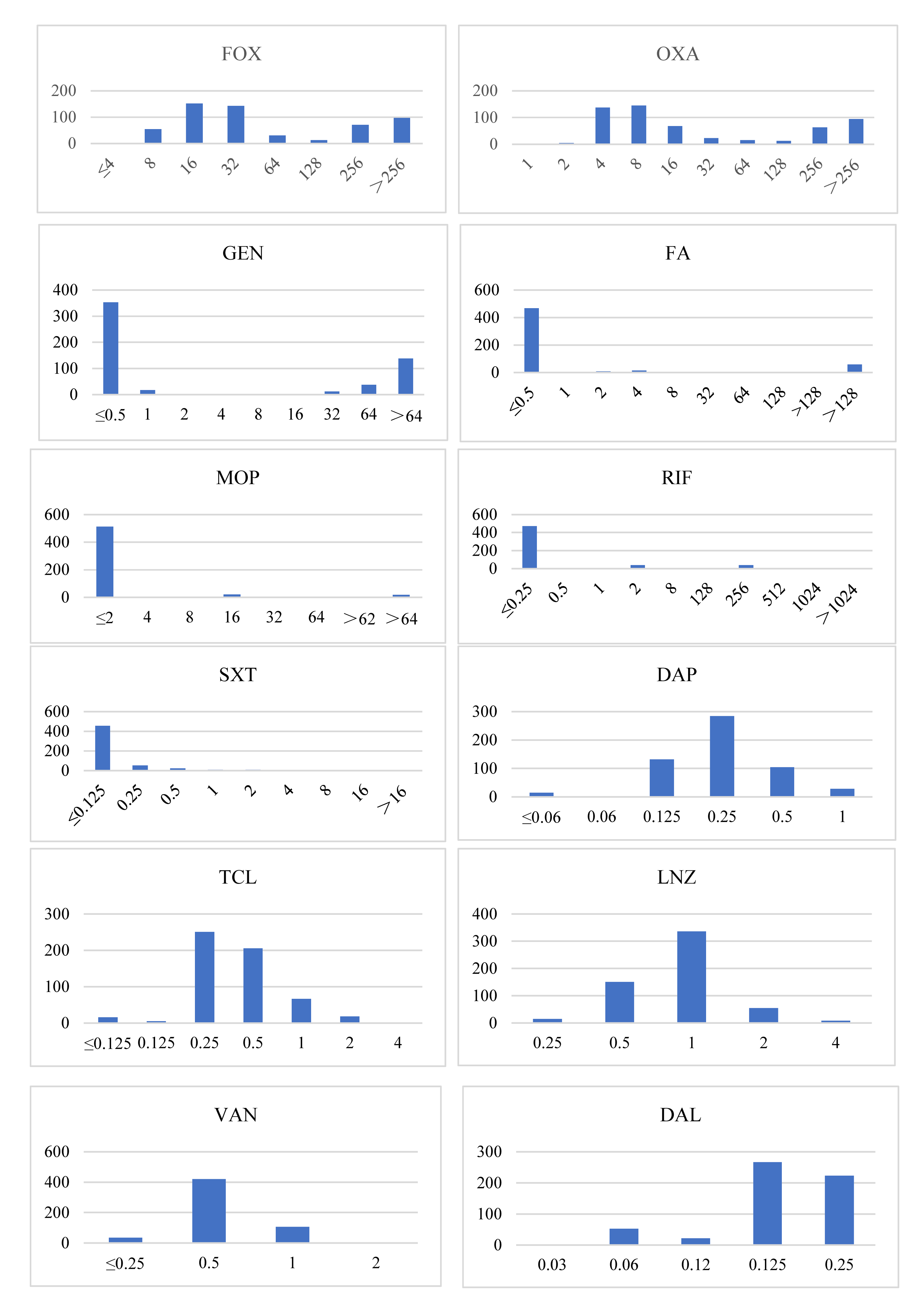


**Figure S1. The MIC distribution of 12 tested antimicrobials for 565 MRSA isolates.** FOX, cefoxitin; OXA, oxacillin; GEN, gentamicin; FA, fusidic acid; MOP, mupirocin; RIF, rifampicin; SXT, sulfamethoxazole/trimethoprim; DAP, daptomycin; TCL, teicoplanin; LNZ, linezolid; VAN, vancomycin; DAL, dalbavancin.
