# Supplementary figures and images for: Methicillin-resistant Staphylococcus aureus in China: a multicentre longitudinal study and whole-genome sequencing
Source: Emerg Microbes Infect. 2022 Feb 10;11(1):532–42. doi: 10.1080/22221751.2022.2032373 (PMC8843102; doi:10.1080/22221751.2022.2032373)

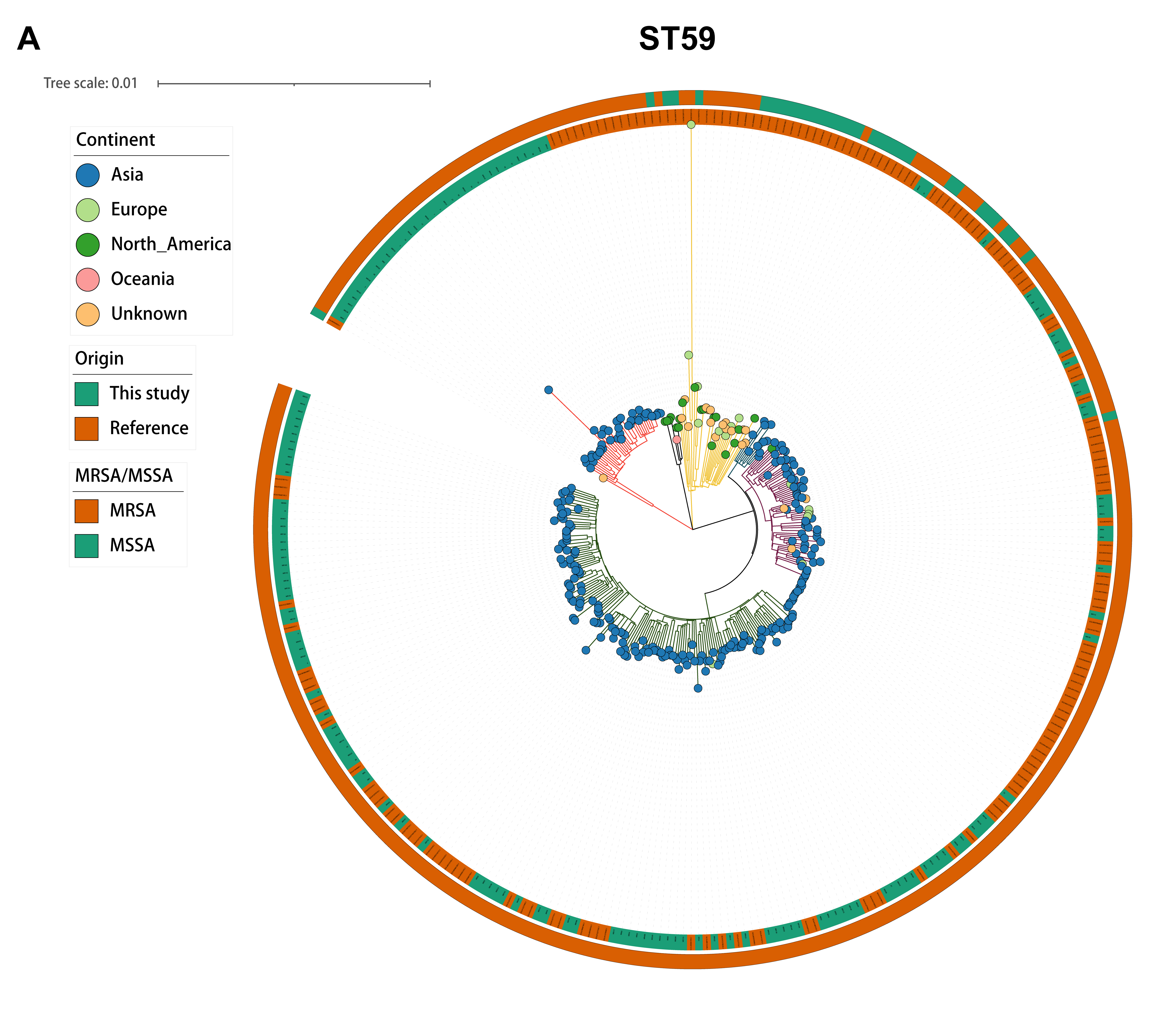

Supplement: Supplemental Material [file TEMI_A_2032373_SM2438.zip › Suppl files/Supplemental file 3 Figure S2A.docx]

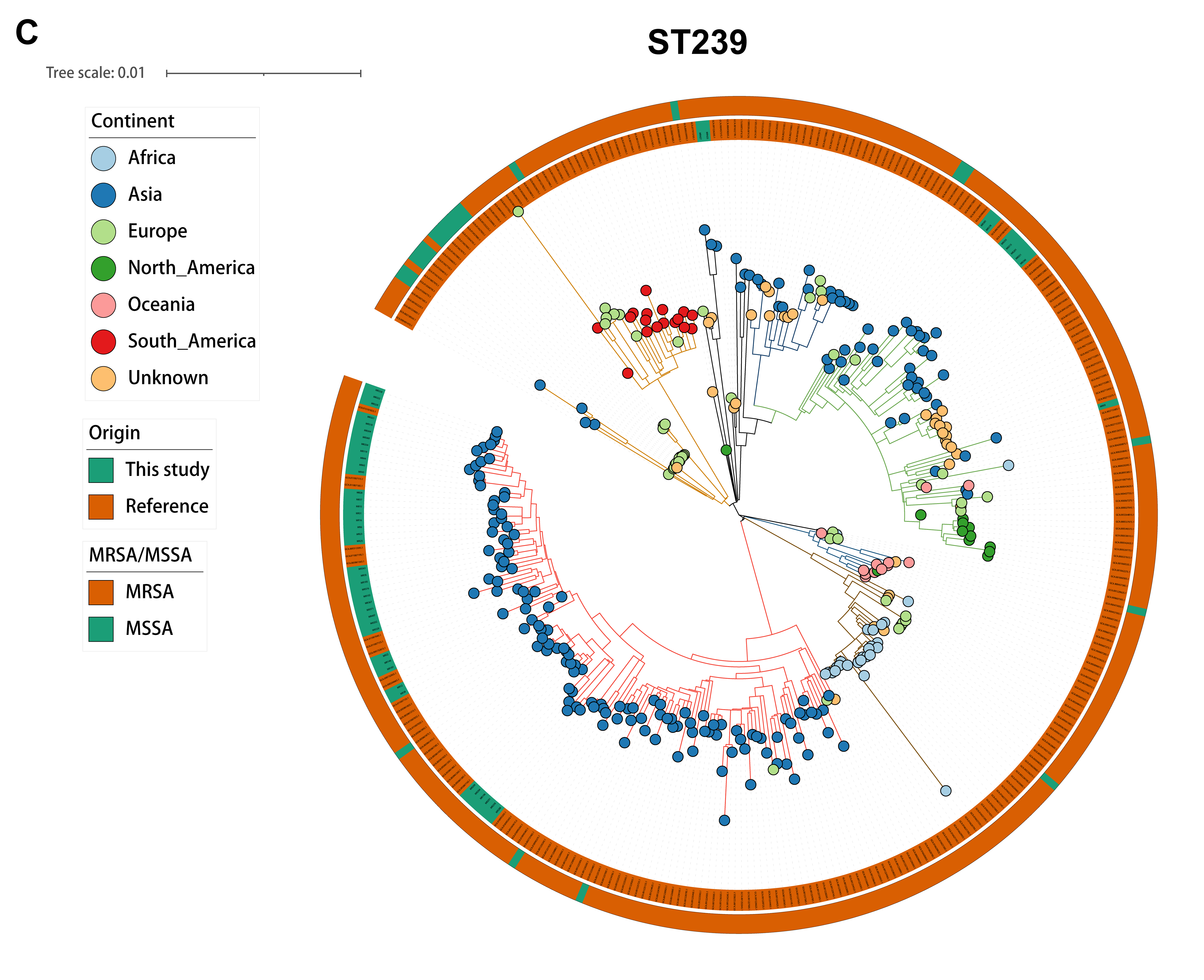

Supplement: Supplemental Material [file TEMI_A_2032373_SM2438.zip › Suppl files/Supplemental file 3 Figure S2C.docx]

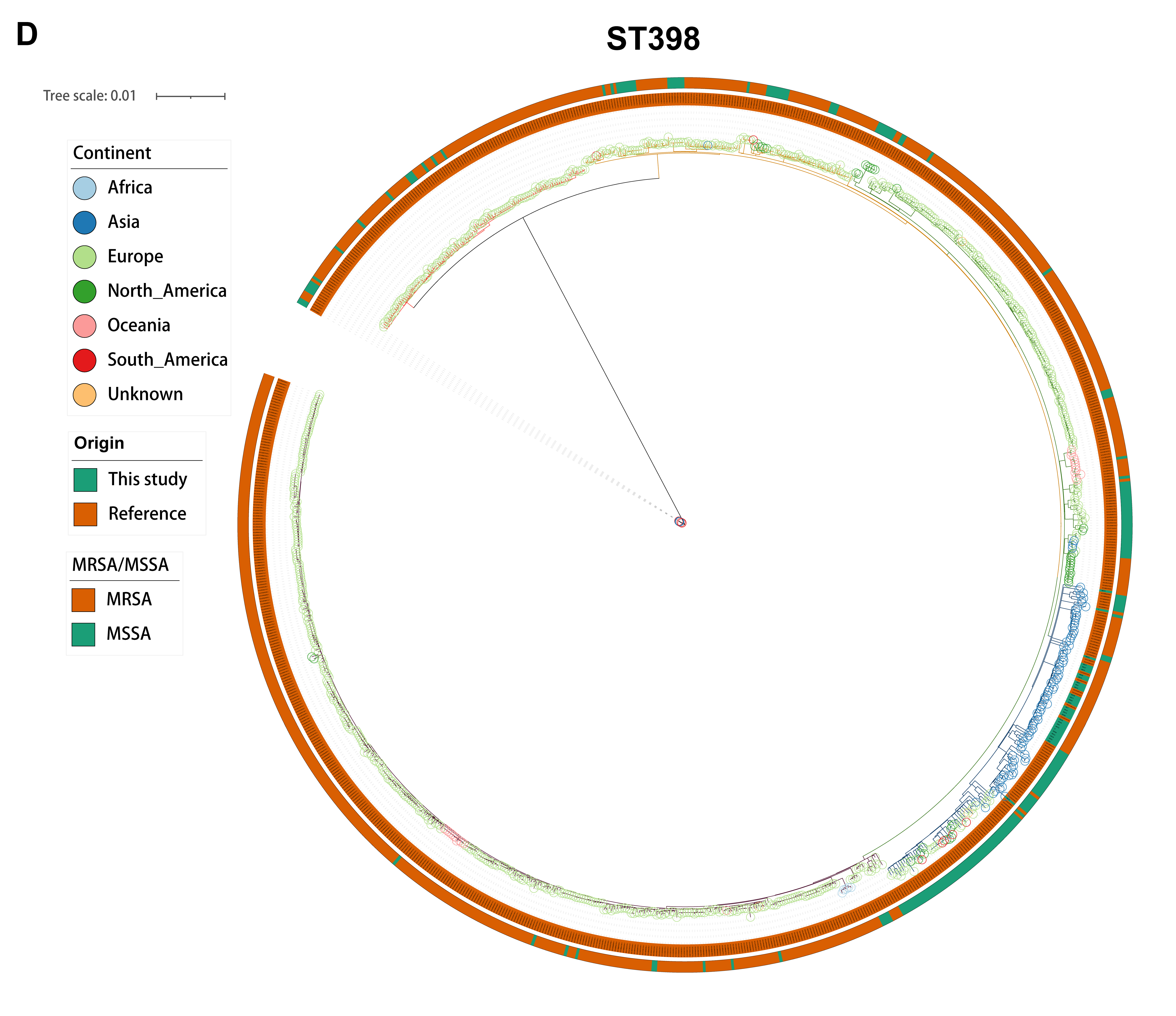

Supplement: Supplemental Material [file TEMI_A_2032373_SM2438.zip › Suppl files/Supplemental file 3 Figure S2D.docx]
